# Prediction of Molecular Mutations in Diffuse Low-Grade Gliomas using MR Imaging Features

**DOI:** 10.1038/s41598-020-60550-0

**Published:** 2020-02-28

**Authors:** Zeina A. Shboul, James Chen, Khan M. Iftekharuddin

**Affiliations:** 1 Vision Lab, Electrical & Computer Engineering, Old Dominion University, Norfolk, VA USA; 2 University of California San Diego Health System, San Diego, CA USA; 3 Department of Radiology, San Diego VA Medical Center, San Diego, CA USA

**Keywords:** Cancer imaging, Molecular medicine

## Abstract

Diffuse low-grade gliomas (LGG) have been reclassified based on molecular mutations, which require invasive tumor tissue sampling. Tissue sampling by biopsy may be limited by sampling error, whereas non-invasive imaging can evaluate the entirety of a tumor. This study presents a non-invasive analysis of low-grade gliomas using imaging features based on the updated classification. We introduce molecular (MGMT methylation, *IDH* mutation, 1p/19q co-deletion, *ATRX* mutation, and *TERT* mutations) prediction methods of low-grade gliomas with imaging. Imaging features are extracted from magnetic resonance imaging data and include texture features, fractal and multi-resolution fractal texture features, and volumetric features. Training models include nested leave-one-out cross-validation to select features, train the model, and estimate model performance. The prediction models of MGMT methylation, *IDH* mutations, 1p/19q co-deletion, *ATRX* mutation, and *TERT* mutations achieve a test performance AUC of 0.83 ± 0.04, 0.84 ± 0.03, 0.80 ± 0.04, 0.70 ± 0.09, and 0.82 ± 0.04, respectively. Furthermore, our analysis shows that the fractal features have a significant effect on the predictive performance of MGMT methylation *IDH* mutations, 1p/19q co-deletion, and *ATRX* mutations. The performance of our prediction methods indicates the potential of correlating computed imaging features with LGG molecular mutations types and identifies candidates that may be considered potential predictive biomarkers of LGG molecular classification.

## Introduction

Diffuse low-grade gliomas (LGG) are World Health Organization (WHO) Grade II and III gliomas. They are infiltrative in their nature and arising from glial cells (astrocytes or oligodendrocytes) of the central nervous system (CNS)^1,2^. Recurrence and malicious progression are possible because of the difficulty in complete tumor resection^3^. A group of these tumors may also develop into glioblastoma (GBM).

An updated classification of diffuse LGG was included in the 2016 WHO Classification of Tumors of the CNS^4^. The new classification of the diffuse LGG depends on the genetic driver mutations (*IDH* mutations, 1p/19q co-deletion, *TERT* mutations, and *ATRX* mutations). This new classification correlates well with patients’ treatment and survival, for example, oligodendroglioma, defined by the 1p/19q co-deletion, are associated with longer survival compared to astrocytoma, which do not harbor the 1p/19q co-deletion^5^.

Molecular mutations are determined using invasive methods by obtaining usable tissue samples that have an increase in proliferation and neovascularization^6^. Tissue sampling may also be associated with high cost, morbidity, and even mortality^7^, and depending on the sample, may undersample tumor components, especially in heterogeneous tumors. Consequently, developing alternative methods and non-invasively classify diffuse LGG into its different subtypes using imaging features and machine learning techniques have emerged as a promising body of research. In this work, we propose a non-invasive imaging-based classification of diffuse LGG using molecular mutations and histology prior to invasive tissue sampling.

Isocitrate Dehydrogenase mutations, *IDH1*, and *IDH2* have been found in gliomas^8,9^, and classifying gliomas based on their molecular profiling of *IDH* status (mutated vs. wild-type) creates clinically distinct groups. *IDH* wild-type gliomas behave aggressively when compared with the *IDH* mutant gliomas. As a result, patients with *IDH* mutant gliomas tend to have better prognosis^10^. A 1p/19q co-deletion is considered as a molecular marker of oligodendroglioma and is associated with *IDH* mutation^11^ and improved survival^8^. This genetic alteration happens when the short arm of chromosome 1 (1p), and the long arm of chromosome 19 (19q) are deleted. Another mutation that is strongly associated with 1p/19q co-deletion is the mutations in the promoter region of the telomerase reverse transcriptase (*TERT*)^12^. *TERT* mutation is associated with poor survival in the absence of *IDH* mutation^13^. *ATRX* is a somatic mutation in the Alpha-Thalassemia/mental Retardation syndrome known as X-linked and may be reported in gliomas including GBM^14^ and is associated with a significantly better prognosis^15,16^. In addition, *ATRX* mutation often occurs with *IDH* mutations and is almost mutually exclusive with 1p/19q co-deletion. Another molecular alteration that has a high prevalence of LGG is O6-methylguanine-DNA methyltransferase (MGMT) gene promoter methylation^17^. Patients with a methylated MGMT promoter are associated with better overall survival^18,19^. MGMT promoter has a better impact on overall survival if MGMT methylation is combined with *IDH* mutation and 1p/19q co-deletion^18^.

Diffuse LGG is known for its heterogeneous characteristic that reveals variances in tumor biology. This heterogeneity can be seen through the histological types: astrocytoma, oligoastrocytoma, and oligodendroglioma^4,20^, although oligoastrocytoma is no longer used when molecular markers are available. The heterogeneity can be characterized by magnetic resonance imaging (MRI) features^21–23^, which suggests using MRI features as a non-invasive marker in tumor grading and classification^24–28^.

Our study addresses diffuse LGG grading and classification prediction based on molecular mutations using imaging features that are extracted from multimodality raw MRI sequences (T1, contrast-enhanced T1(T1 Gd), T2 FLAIR, and T2) of the anatomically depicted tumor volume, and texture representations of the tumor MRI sequences. The extracted features describe the multi-resolution texture, texture features, volumetric, and area-based characteristics. In this study, different molecular (*IDH*, 1p/19q co-deletion, *ATRX*, and *TERT*), and MGMT methylation prediction models are introduced. In addition, our study investigates the efficacy of our novel texture features the fractal and multi-resolution fractal modeling on the performance of the non-invasive prediction of molecular mutation in LGG.

Few studies have shown association between different types of imaging features such as the grey-level co-occurrence matrix (GLCM) for texture, volume and area related features, and intensity-based features to the tumor classification^29–32^. While GLCM features may capture the grey-level spatial variation in an image, these deterministic features may not be effective in analysis of the random surface structure variation of abnormal tumor tissues in MRI. Wavelet features, on the other hand, examine the intensity variation of the tumor tissues in different image resolutions^33,34^. In comparison, the multi-resolution fractal modeling mathematically combines the capabilities of regular texture analysis (e.g., GLCM) and multi-resolution analysis (e.g., wavelets) and, hence, may capture the randomly varying complex structure of the tumor tissue texture at different scales. The spatial intensity distributions of abnormal brain tissues in MRI have a degree of randomness that are amenable to fractal and multi-resolution fractal texture modeling. Several studies have shown the efficacy of fractal and multi-resolution fractal feature analysis for characterization, segmentation and classification of the complex abnormal brain tissues in MRI^35–38^.

Consequently, in this study, we hypothesize that the fractal and multi-resolution fractal modeling may relate to the underlying structure of molecular mutations. To the best of our knowledge, this is the first study that addresses the potency of fractal and multi-resolution fractal features in molecular mutations prediction.

## Material and Methods

### Dataset

In this study, we use a total of 108 pre-operative LGG patients described in^39–41^. Four sequences of the MRI are provided with the data set: pre-contrast T1-weighted (T1), post-contrast T1-weighted (T1Gd), T2-weighted (T2), and T2 Fluid Attenuated Inversion Recovery (FLAIR). These scans are skull-stripped, re-sampled to $1\,{{\rm{mm}}}^{3}$ resolution, and co-registered to the T1 template. The dataset provides the segmented sub-regions of the LGG: Gadolinium enhancing tumor (ET), the peritumoral edema (ED), and the necrosis along with non-enhancing tumor (NCR/NET).

Molecular alterations (*IDH* mutation, 1p/19q co-deletion, *ATRX*, and *TERT* mutation), grade (II and III), and clinical data are downloaded from the Genomic Data Commons Data Portal (https://portal.gdc.cancer.gov/). Clinical data are de-identified by the Health Insurance Portability and Accountability Act of 1996 (HIPAA). The distribution of the data is as follows: (i) *IDH* mutation: 85 Mutant (of which 27 cases are co-deleted) and 23 wild-type (WT), (ii) 1p/19q co-deletion: 27 co-deletion and 81 non-co-deletion, (iii) *ATRX* status: 43 Mutant and 65 WT, (iv) *TERT* status: 46 Mutant and 62 WT, and (v) O6-methylguanine-DNA methyltransferase (MGMT) promoter methylation: 91 methylated and 14 un-methylated. The range of the patients’ age at the diagnosis is 20–75 years and the median are 46.5 years.

### Methodology

In this study, we introduce different molecular prediction models based on fractal and multi-resolution fractal texture features and other MR imaging features. These molecular models include the *IDH*, 1p/19q co-deletion, MGMT, *ATRX*, and *TERT* prediction. A classical way to avoid overfitting is to divide the dataset into training, validation and testing datasets^42^. The dataset is randomly partitioned into *n* pairs (partitions) of training (75% of the entire dataset = 81 cases) and testing (25% of the entire dataset = 27 cases). A balanced distribution of the target molecular mutation is ensured in the training and testing sets in each molecular prediction model. The features are extracted from multimodality MRI sequences of the tumor volume in the training partition. Then, a recursive feature selection is performed to select the number of features and validated with Leave-One-Out Cross-Validation (LOOCV). The selected features are then trained using an Extreme Gradient Boosting (XGBoost) method along with LOOCV. Then, a prediction performance is evaluated using the testing partition.

**Figure 1:**
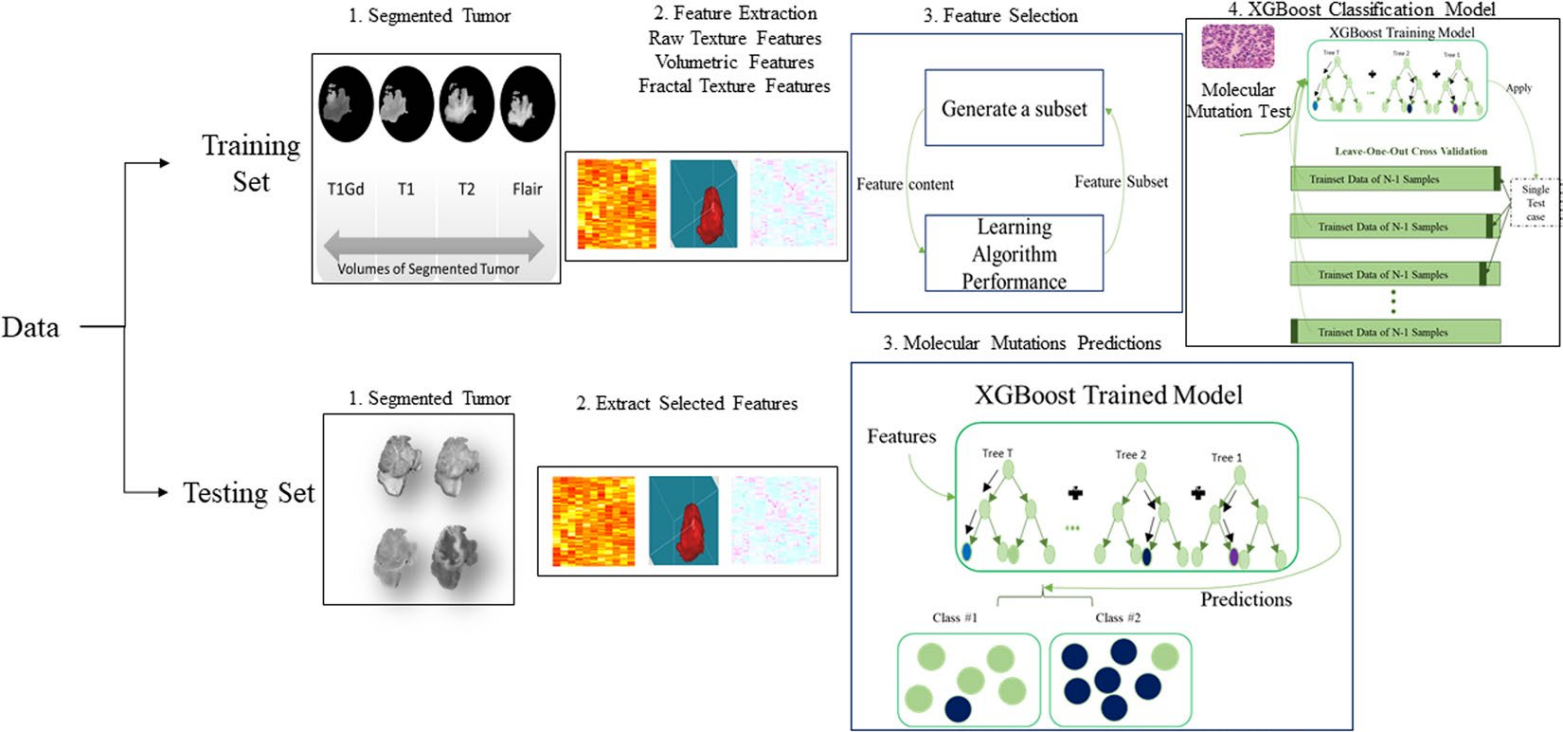
The general outline of the LGG-XGBoost molecular prediction model. Note that this analysis is repeated *n* times (randomly chosen) to generate a more reliable performance.

Furthermore, we study the efficacy of fractal and multi-resolution fractal texture features (e.g., piecewise-triangular prism surface area (PTPSA), multi-resolution fractional Brownian motion (mBm), and Holder Exponent (HE)) extracted from tumor volumes on the performance of the molecular mutation prediction models. Figure 1 shows the overall pipeline of the proposed LGG-XGBoost prediction model for different molecular mutations in LGG.

#### Feature extraction

In this study, around 680 features are extracted to represent texture, volume, and area of the tumor and its sub-regions (edema, enhancing tumor, and necrosis). These features include 41 texture features^43^ extracted from the tumor volume in raw MRI (T1Gd, T2, and FLAIR) sequences and an additional three different texture characterizations of the tumor region. The three texture characterizations are as follows:

- fractal characterization using our PTPSA^44^ modeling,
- multi-resolution mBm^36^ modeling,
- and the characterization Holder Exponent (HE)^45^ modeling of the tumor region.

The computational algorithm of the PTPSA, mBm, and the HE is found in^36,44,46^. Furthermore, six histogram-based statistics (mean, variance, skewness, kurtosis, energy, and entropy) features are also extracted from the different tumor sub-regions (edema, enhancing tumor, and necrosis), respectively.

In addition, we extracted 12 volumetric features: the volume of the whole tumor, the volume of the whole tumor with respect to the brain, the volume of sub-regions (edema, enhancing tumor, and necrosis) divided by the whole tumor, the volume of sub-regions (edema, enhancing tumor, and necrosis) divided by the brain, the volumes of the enhancing tumor and necrosis divided by the edema, the summation of the volume of the edema and enhancing tumor, the volume of the edema divided by the summation of the volume of enhancing tumor and necrosis, and the volume of the necrosis divided by the summation of the volume of the edema and enhancing tumor. Finally, nine-volume properties (area, bounding box, centroid, perimeter, major axis length, minor axis length, eccentricity, orientation, solidity, and extent) are extracted from the tumor volume and from three viewpoints (*x*, *y*, and *z*-axes) of the tumor and its sub-regions (edema, enhancing tumor, and necrosis).

Texture features are analyzed using MATLAB-based software developed by Vallières *et al*.^43^. Fractal characterization, multi-resolution fractal characterization, HE characterization, and volumetric features are analyzed using MATLAB-based in-house software.

#### Molecular mutation prediction model and feature selection

The molecular mutation prediction model is performed on the training set using nested LOOCV to avoid an optimistic performance estimate. Recursive feature selection is performed in the inner loop to find optimum features set. In each mutation prediction analysis, feature selection using RFS is done using the training set that is (75%) of the whole 108 cases. Because we repeat the prediction analysis of training and testing n times, eventually each case of the 108 cases would have appeared in the training sets. The LOO cross-validated performance of the molecular mutation prediction model is estimated in the outer loop. The molecular mutation prediction model is performed using the R statistical packages Caret and XGBoost (www.r-project.org).

Recursive feature selection (RFS) is implemented by first fitting a Random Forest (RF) model to all features. Each feature is ranked by its importance, and the least important features are removed from the current feature set. Then, this step is repeated recursively until the optimum features set that has the best performance is reached. In our implementation of recursive feature selection, the number of features in the features’ sets are 2, 3, 5, 7, 9, 11, 13, and 15 features. In addition, the best performance is determined by maximizing the area under the receiver operating characteristic (ROC) metric. The features’ set that provides the combination of features that maximize the area under the ROC (AUC) is chosen for training in the prediction model. Using recursive feature selection, the maximum number of selected features is fifteen, so that the training samples (eighty-one cases) are at least 5 times the number of features to reduce model overfitting.

In our study, XGBoost is utilized as a classification and prediction model using the optimum features set as input and molecular mutation information as the target output. XGBoost^47^ is an advanced tree boosting supervised machine learning technique that is effective in handling imbalanced datasets. XGBoost is widely used in classification and regression tasks. For a given dataset *D* with *s* samples and *m* features $D=\{({x}_{i},{y}_{i})\}(|D|=s,\,{x}_{i}\in {{\mathbb{R}}}^{m},\,{y}_{i}\in {\mathbb{R}})$, a tree ensemble model uses *K* additive functions to predict the output as follows,

1 $$\widehat{{y}_{i}}=\mathop{\sum }\limits_{k=1}^{K}\,{f}_{k}({x}_{i}),\,{f}_{k}\in F,$$

where ${x}_{i}$ is the feature/input vector, ${y}_{i}$ is the target/output variable, and $f(x)$ is a function in the functional space $F$, and $F$ is a set of all possible classification and regression trees. One of the major advantages of using XGBoost is that XGBoost provides L1 and L2 regularization. L1 regularization handles sparsity, whereas L2 regularization reduces overfitting. In addition, we choose XGBoost because it is known for handling an imbalanced dataset. A detailed mathematical derivation of the XGBoost algorithm is found in Chen *et al*.^47^.

The final molecular mutation prediction model (that is used for the testing set) is obtained by fitting the optimum features’ set that maximize the performance in the inner loop (over all the outer cross-validation loops). Note, if there are more than one feature sets maximizes the inner loop performance, then the common feature between the features’ sets are used. The prediction performance of the final molecular mutation model is tested using the paired testing sets (partitions).

Finally, in order to study the efficacy of fractal and multi-resolution fractal texture features used in this study (e.g., PTPSA, mBm, and Holder Exponent) on the performance of the proposed prediction models as shown in Fig. 1, we perform molecular prediction analyses with and without these texture features, respectively. The whole process in Fig. 1 is repeated *n* times independently with *n* different training/testing set pairs. The *n* number of repetitions is a random number between 10 and 15 that are generated for each model.

#### Evaluation

The molecular models are validated using separate testing sets and the prediction performance (test performance) of the trained XGBoost model is estimated using AUC, sensitivity, and specificity. After *n* times of independent repetitions, ANOVA test is used to compare the difference in the prediction performance between two models with and without the fractal and multi-resolution fractal texture in the prediction models. In addition, ANOVA is used to analyze the significant association between features and the different molecular mutations. The survival groups that are formed using the significant features are compared using Kaplan-Meier curves and the log-rank test. The hazard ratio of features is determined using the Cox proportional hazards model and assessed using the likelihood-ratio test. Finally, the evaluation step for Survival is conducted using R statistical packages.

## Results

Around 680 imaging features are extracted from multimodality MRI sequences. Recursive feature selection is used to find the optimum number of significant features for each molecular mutation prediction model. Our analysis of the different prediction models are repeated independently *n* times with different training and testing pairs (partitions). Table 1 displays the number of repetitions *n*, LOOCV performance, and the test performance of the different prediction models when including/removing texture characterization of the fractal and multi-resolution fractal of PTPSA, mBm, and Holder Exponent characterization.

**Table 1:** LOO cross-validated performance of the outer-loop, and the predictive/test performance of the different LGG molecular prediction models.

Cross-Validated performance	*n* repetition	With fractal & multi-resolution fractal features			Without fractal & multi-resolution fractal features		
		AUC.	Sens.	Spec.	AUC.	Sens.	Spec.
MGMT Methylation	11	0.86 ± 0.03	0.88 ± 0.02	0.80 ± 0.09	0.87 ± 0.04	0.90 ± 0.02	0.66 ± 0.10
*IDH* mutation	13	0.85 ± 0.04	0.90 ± 0.03	0.75 ± 0.05	0.79 ± 0.07	0.89 ± 0.08	0.75 ± 0.07
1p/19q co-deletion	15	0.83 ± 0.03	0.78 ± 0.08	0.83 ± 0.03	0.80 ± 0.05	0.63 ± 0.08	0.87 ± 0.02
*ATRX* mutation	10	0.77 ± 0.06	0.62 ± 0.09	0.80 ± 0.03	0.80 ± 0.04	0.77 ± 0.06	0.76 ± 0.03
*TERT*	14	0.82 ± 0.04	0.70 ± 0.06	0.83 ± 0.04	0.82 ± 0.04	0.82 ± 0.04	0.76 ± 0.05
**Prediction/test Performance**							
MGMT Methylation	11	0.83 ± 0.04	0.93 ± 0.05	0.73 ± 0.13	0.70 ± 0.12	0.90 ± 0.07	0.50 ± 0.24
*IDH* mutation	13	0.84 ± 0.03	0.90 ± 0.06	0.79 ± 0.09	0.75 ± 0.07	0.83 ± 0.11	0.66 ± 0.18
1p/19q co-deletion	15	0.80 ± 0.04	0.75 ± 0.08	0.85 ± 0.06	0.75 ± 0.07	0.67 ± 0.12	0.84 ± 0.10
*ATRX* mutation	10	0.70 ± 0.09	0.69 ± 0.06	0.83 ± 0.10	0.66 ± 0.10	0.65 ± 0.16	0.68 ± 0.18
*TERT*	14	0.82 ± 0.04	0.77 ± 0.12	0.86 ± 0.09	0.78 ± 0.07	0.77 ± 0.11	0.79 ± 0.11

### MGMT methylation model

**Figure 2:**
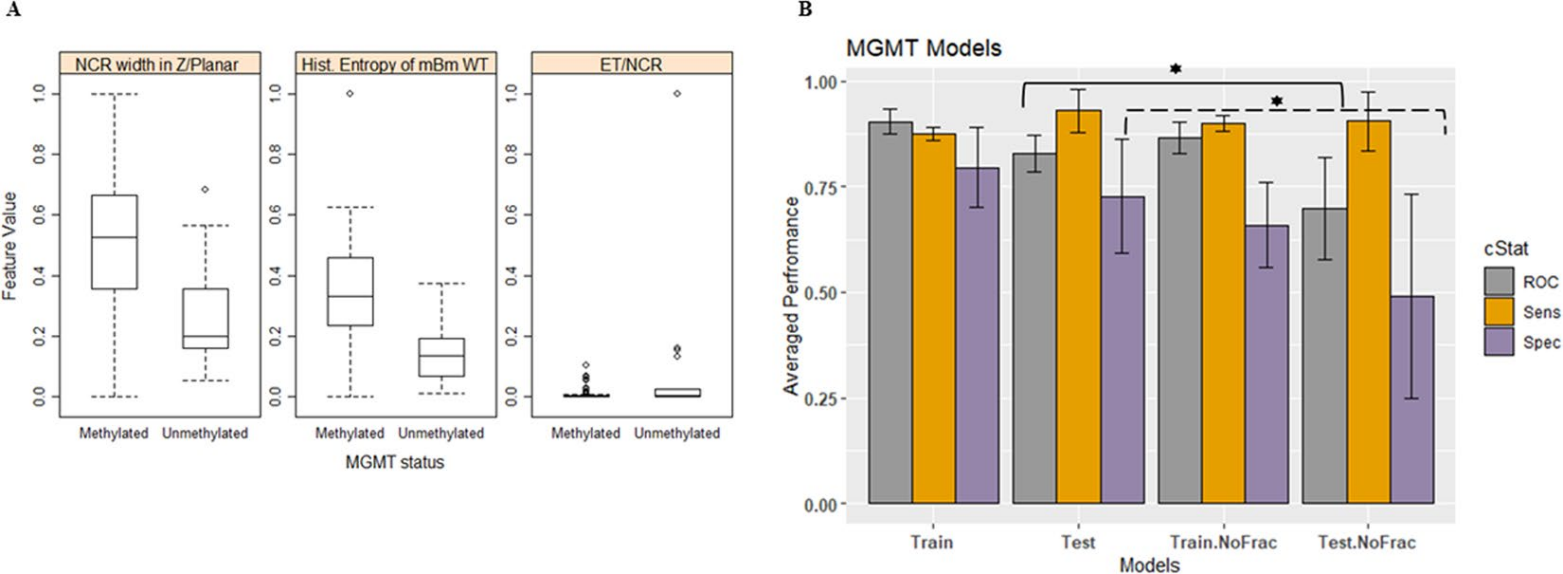
MGMT methylation models. (**A**) Distribution of the most selected features in discriminating MGMT mutated and WT, and (**B**) MGMT prediction model performance using the train and test partitions with and without fractal texture features. Error bars represent two standard deviations. The asterisk *illustrates the significant difference between the two measurements.

The most frequent features that are selected are the necrosis width in the *Z* planar, the histogram entropy of the mBm characterization on the whole tumor, and size ratio between the enhancing tumor and the necrosis. The necrosis width in the *Z* planar and the histogram entropy of the mBm characterization on the whole tumor features are significantly (ANOVA test, *p*-value < 0.05) associated with methylated MGMT, whereas the size ratio between enhancing tumor and necrosis is associated significantly with un-methylated MGMT as shown in Fig. 2A.

The LOOCV performance and the prediction performance on the testing set for predicting the MGMT methylation status using imaging features are illustrated in Table 1 and Fig. 2B. The prediction performance using the test partitions achieves an AUC, a sensitivity, and a specificity of 0. 0.83 ± 0.04, 0.93 ± 0.05, and 0.73 ± 0.13, respectively. Removal of our fractal and multi-resolution fractal features from MGMT methylation prediction model have an effect on the prediction performance on the testing set. AUC and specificity drop significantly (ANOVA test, *p*-value = 0.003, and 0.01, respectively) when the fractal features are removed (Fig. 2B).

### *IDH* mutation model

**Figure 3:**
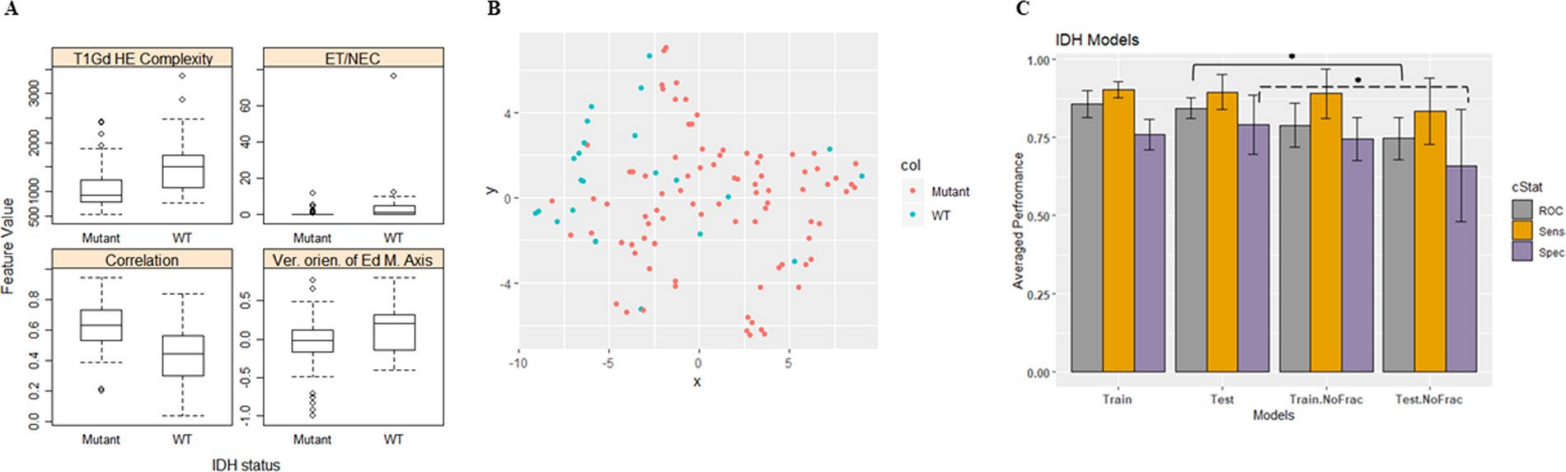
IDH models. (**A**) Distribution of the most selected features in discriminating among IDH mutated and WT cases in LGG, (**B**) 2-D t-Distributed Stochastic Neighbor Embedding (t-SNE) visualization using only the 4 features in (**A,C**) Performance comparison of IDH prediction model using the train and test partitions with and without fractal features. Error bars represent two standard deviations. The asterisk *illustrates the significant difference between the two measurements.

Our analysis reveals that the tumor correlation, the vertical orientation of edema major axis (the angle between the edema major axis and the vertical axis), size ratio between the enhancing tumor and the necrosis, and the complexity of holder exponent of the tumor are among the most frequently selected features to predict *IDH*-mutated status in LGG. Higher values of the complexity of the holder exponent of the tumor, the size ratio between the enhancing tumor and the necrosis, and higher values of the vertical orientation of edema major axis associate significantly (ANOVA test, *p*-value < 0.005) with WT *IDH* status. Whereas the tumor correlation associates significantly (ANOVA test, *p*-value < 0.005) with mutated *IDH* status as illustrated in Fig. 3A. Figure 3B shows the clustering of IDH status using the most frequent features in Fig. 3A. The clustering between the mutated *IDH* and WT *IDH* is demonstrated using t-Distributed Stochastic Neighbor Embedding^48^ (tSNE).

In addition, the tumor correlation, the vertical orientation of edema major axis, and the complexity of holder exponent of the tumor features carry a hazard ratio [HR] of 0.562 (95% CI, 0.381–0.828), 2.655 (95% CI, 1.617–4.36), and 1.553 (95% CI, 1.165–2.07) with a likelihood ratio test *p*-value = 0.005, 0.0001, 0.008, respectively. Because these features are continuous features, the HRs interpolate as follows: the risk of death increases (or decreases if HR < 1) by (HR-1) × 100% for every 1-standard deviation increase in that feature. The LOOCV and the test performance of the proposed IDH models are illustrated in Table 1. The prediction performance using the testing partitions achieves an AUC, sensitivity, and specificity of 0.84 ± 0.03, 0.90 ± 0.06, and 0.79 ± 0.09, respectively. Note that the AUC and specificity of the IDH status prediction model drop significantly to 0.75 ± 0.07 and 0.66 ± 0.18 (ANOVA test, *p* = 0.0001 and *p* = 0.028, respectively) after removing features extracted from fractal and the multi-resolution modeling (Fig. 3C).

### 1p/19q co-deletion model

**Figure 4:**
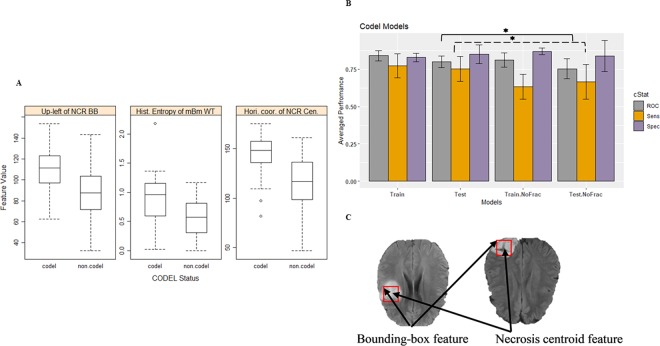
1p/19q codeletion models. (**A**) Distribution of the most selected features in discriminating 1p/19q codeletion and non-codeletion, (**B**) Performance comparison of codeletion classifier models using the train and test partitions with and without fractal features. (**C**) Example of FLAIR images illustrates the location of the necrosis centroid and the upper-left location of the necrosis bounding-box. Error bars represent two standard deviations. The asterisk *illustrates the significant difference between two measurements.

The necrosis upper-left bounding box location, the histogram entropy of mBm characterization of the whole tumor, and the horizontal coordinate of necrosis centroid are the most frequent optimum features that are selected in our proposed 1p/19q codeletion models. These three features show significance (ANOVA test, *p*-value < 0.0001) associated with the existence of the 1p/19q co-deletion. Our analysis shows frontal tumors are associated significantly (ANOVA test, *p*-value < 0.0001) with 1p/19q co-deletion mutations (Fig. 4A). The performance of the proposed co-deletion prediction LOOCV model is illustrated in Table 1. The 1p/19q co-deletion performance using the test partitions achieve an AUC of 0.80 ± 0.04, a sensitivity of 0.75 ± 0.08, and a specificity of 0.85 ± 0.06. In addition, the efficacy of our fractal and multi-resolution fractal texture features on the performance of the co-deletion prediction model is significant as shown in (Fig. 4B and Table 1). The AUC and the sensitivity of the co-deletion prediction model drop significantly (ANOVA test, *p*-value of 0.024 and 0.029, respectively) after removing features extracted from our fractal and multi-resolution fractal features in the 1p/19q co-deletion prediction model to 0.75 ± 0.07 and 0.67 ± 0.12 (without fractal features). Figure 4C illustrates the location of the centroid and the upper-left bounding box of the necrosis. The histogram entropy of mBm of the tumor volume offers HR of 0.59 per standard deviation (95% CI, 0.35–0.97) with a likelihood ratio test *p*-value of 0.037.

### *ATRX* Mutation Model

**Figure 5:**
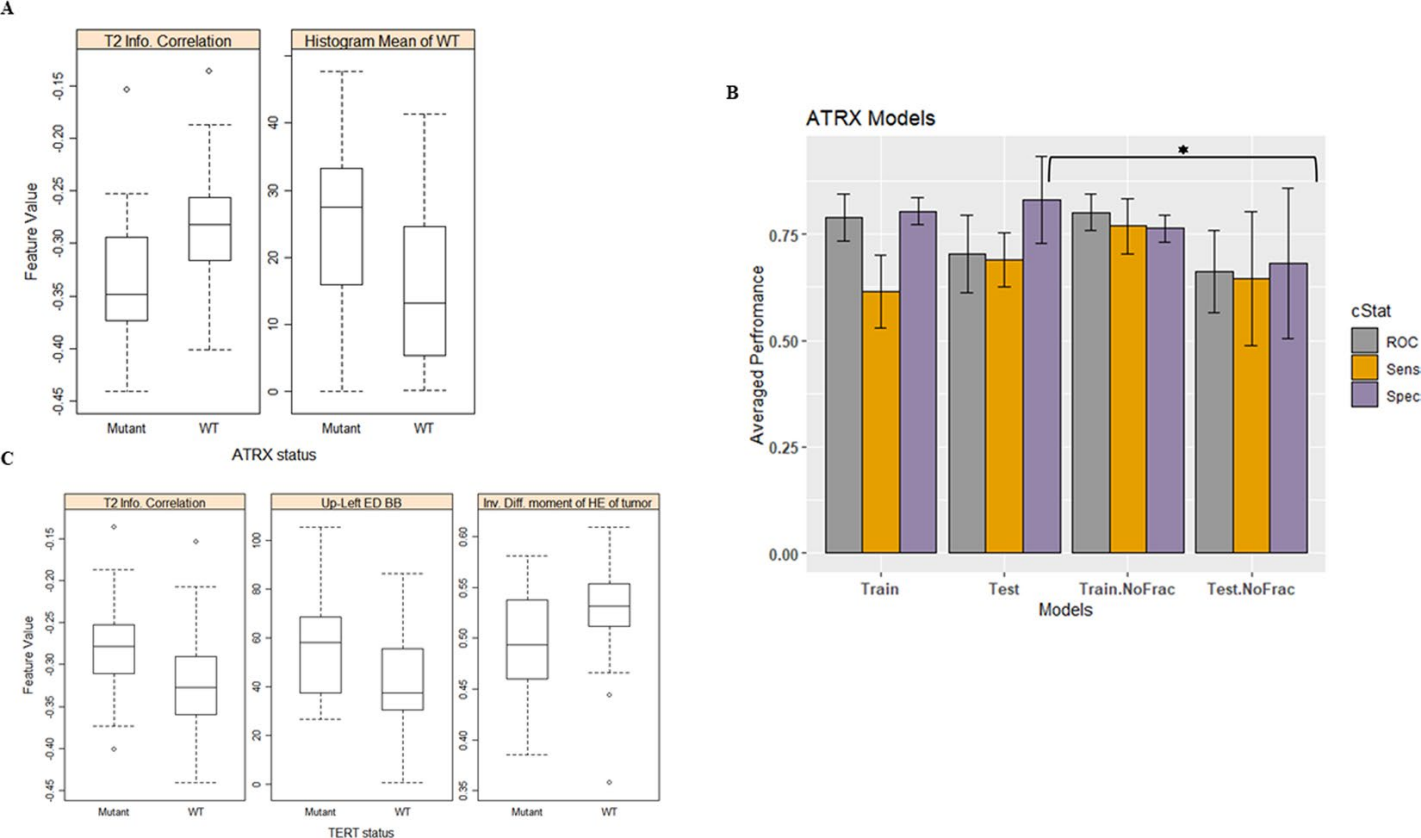
Distribution of the most selected feature in discriminating (**A**) mutated ATRX and WT, (**B**) Performance comparison of *ATRX* classifier models using the train and test partitions with and without fractal features. Error bars represent two standard deviations. The asterisk *illustrates the significant difference between the two measurements, and (**C**) mutated TERT and WT.

The information content of correlation and the histogram mean of the tumor volume of the most frequently selected features are employed in the XGBoost model to train and predict *ATRX* status. The distribution of the most frequent features is illustrated in Fig. 5A. Higher values of information of correlation are associated significantly (ANOVA test, *p*-value < 0.001) with *ATRX* wild-type. Whereas Higher values of histogram mean are associated significantly (ANOVA test, *p*-value < 0.001) with mutated *ATRX*. The *ATRX* prediction model achieves prediction performance of an AUC of 0.70 ± 0.09, a sensitivity of 0.70 ± 0.06, and a specificity of 0.83 ± 0.10 using the test partitions. Removing features extracted from our fractal and multi-resolution fractal modeling from the *ATRX* prediction model has a significance specificity drop to 0.68 ± 0.18 performance of the model with *p*-value = 0.03 (ANOVA test) as shown in Fig. 5B.

### *TERT* mutation model

A review of the most frequently selected imaging features of TERT mutation prediction model, we notice that the information content of correlation of the tumor volume, the edema upper-left bounding box location, and the inverse difference moment of HE characterization of tumor volume are the most frequently selected features (Fig. 5C). The inverse difference moment of HE characterization of tumor volume offers HR of 0.612 per standard deviation (95% CI, 0.405–0.924) with a likelihood ratio test *p*-value = 0.027. The *TERT* prediction models’ performances are illustrated in Table 1. The TERT prediction performance using the test partitions achieves an AUC of 0.82 ± 0.04, a sensitivity of 0.77 ± 0.12, and a specificity of 0.86 ± 0.09, respectively. Removing our fractal and multi-resolution fractal texture modeling has no significant effect on the *TERT* performance of prediction models when using the test partitions as shown in Table 1.

### *IDH*/co-deletion model

Molecular classification based on the status of *IDH* mutations and 1p/19q co-deletion results in distinguishing three LGG molecular subtypes that have a distinct clinical behavior: *IDH* WT, *IDH* mutant with 1p/19q co-deleted, and *IDH* mutant with 1p/19q non-co-deleted. In this work, we combine these two mutations and perform a 3-class prediction (i.e., IDH/co-deletion prediction model) using the methodology illustrated previously and shown in Fig. 1.

The *IDH* mutation model and the 1p/19q co-deletion model show their superior performance when using fractal and multi-resolution fractal modeling features along with other non-fractal (i.e., texture and volumetric) features. Consequently, we develop 3-class *IDH*/co-deletion models utilizing fractal and other non-fractal features. The whole process of training and testing is repeated 5 times. The distribution of the three molecular subtypes is as follows: 23 cases represent *IDH WT*, 27 cases represent *IDH* mutant with 1p/19q co-deleted, and 58 cases represent *IDH* mutant with 1p/19q non-co-deleted.

Our analysis using the Chi-square test confirms a significant association (*p*-value = 0.004) between *IDH* status and 1p/19q codeletion. Additionally, our analysis shows that *IDH WT* patients carry HR of 3.1 (likelihood ratio test *p*-value = 0.007) and have significantly shorter survival when compared to *IDH* mutated patients (19.9 vs. 65.7 months, log-rank test *p*-value = 0.004). The association between *IDH* status and overall survival remains significant after stratifying for 1p/19q codeletion (likelihood ratio test *p*-value = 0.005).

Table 2 shows the performance of the 3-class training and testing of the *IDH*/co-deletion model where the sensitivity and the specificity are reported per class, multiclass AUC is calculated as described in^49^, and the overall accuracy is calculated based on the number of correctly classified in all classes to the total number of cases.

**Table 2:** LOO cross-validated performance of the outer-loop, and the predictive/test performance of the *IDH*/co-deletion model using fractal and multi-resolution fractal features and other non-fractal features.

Cross-Validated performance	Overall Accuracy	Sens.	Spec.	Multiclass AUC
*IDH* WT	0.80 ± 0.01	0.73 ± 0.01	0.93 ± 0.004	0.75 ± 0.06
*IDH* mutation and 1p/19q co-deletion		0.71 ± 0.02	0.90 ± 0.03	
*IDH* mutation and 1p/19q non-co-deletion		0.87 ± 0.03	0.87 ± 0.03	
**Prediction/test Performance**				
*IDH* WT	0.79 ± 0.06	0.73 ± 0.09	0.92 ± 0.03	0.80 ± 0.04
*IDH* mutation and 1p/19q co-deletion		0.71 ± 0.00	0.89 ± 0.05	
*IDH* mutation and 1p/19q non-co-deletion		0.84 ± 0.09	0.85 ± 0.08	

Note this simultaneous prediction of multiple genes may be more clinically valuable when compared to conventional single gene prediction models.

## Discussion

The 2016 WHO classification of diffuse LGGs heavily weighs molecular mutations classifying primary brain tumors with particular importance assigned to *IDH* mutation, 1p/19q co-deletion, *ATRX* mutation, *TERT* mutations, and MGMT methylation. Our study on diffuse LGG is largely able to predict the presence of these important molecular mutations based on MR imaging features. Therefore, prediction of tumor aggressiveness (based on molecular mutations) may be achieved through non-invasive imaging features as an adjunct to traditional visual morphologic diagnosis and invasive tissue sampling.

**Figure 6:**
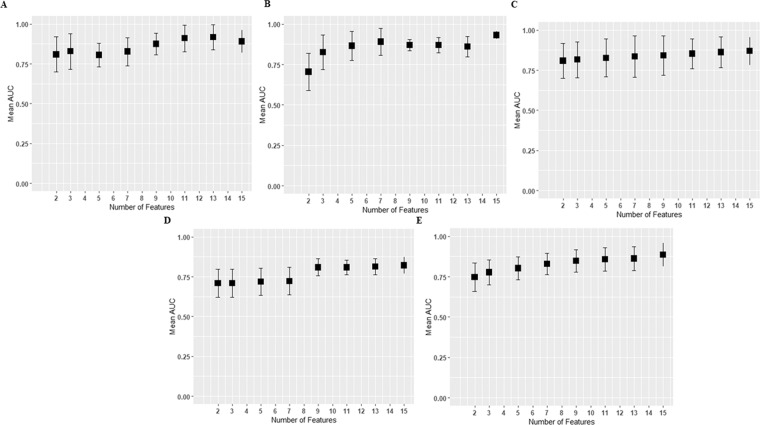
The effect of number of features on cross-validated performance of the different mutation models. (**A**) MGMT prediction model, (**B**) prediction model, (**C**) 1p/19q co-deletion prediction model, (**D**) ATRX prediction model, and **(E**) TERT prediction model. The *y* axis represents mean AUC of every feature set which is computed from all independent *n* repetitions, and error bars represent two standard deviations. The *x* axis represents the number of selected features.

In this work, the number of originally extracted imaging features (six hundred eighty features) is higher than the number of samples (eighty-one cases) in the training dataset, which may cause overfitting. To address possible overfitting, we implement feature selection in the training model that offers a maximum of fifteen selected features. Figure 6 illustrates the effect of the number of features on the cross-validated performance of the different mutation prediction models. The average AUC performances and the standard error of the different prediction models improve when the number of features is greater than 9. Note that the standard error mostly plateaus when the number of features varies between 9–15 (standard error reflects instability).

For the fractal and multi-resolution fractal texture model in Table 1, the AUC predictive performance of MGMT, 1p/19q co-deletion, and *ATRX* models drop to 0.83 ± 0.04, 0.80 ± 0.04, and 0.70 ± 0.09, respectively. This statistically non-significant drop in performance (ANOVA test, *p* = 0.076, *p* = 0.073, and *p* = 0.071 respectively) when compared to their AUC cross-validated performance, may suggest minimal overfitting for these models. The AUC predictive performances of *IDH* and *TERT* models are almost comparable to their cross-validated performance as shown in Table 1 that suggests there is no overfitting in these two models.

For the non-fractal models in Table 1, the AUC predictive performance of *IDH*, 1p/19q co-deletion, and *TERT* models drop to 0.75 ± 0.07, 0.75 ± 0.07, and 0.78 ± 0.07 respectively. This statistically non-significant drop (ANOVA test, *p* = 0.182, *p* = 0.056, and *p* = 0.062 respectively) when compared to their AUC cross-validated performance, may suggest minimal overfitting for these models. However, the poor predictive performances of the non-fractal MGMT and non-fractal ATRX models when compared with their non-fractal cross-validated performances are a sign of overfitting.

Note “optimally chosen” features in the non-fractal models (in Figs. 2–4) are not selected by simply replacing the fractal features by alternate features (or by using the same number of predictors as in the fractal models). In each mutation model (fractal or non-fractal), RFS (and thus selecting “optimally chosen” features) is performed independently. In our implementation of RFS for fractal or non-fractal models in Figs. 2–4, the possible number of features in the features’ sets may be 2, 3, 5, 7, 9, 11, 13, and 15, respectively. The maximum number of selected features is set to 15 such that the training samples (81 cases) are at least 5 times the number of features to reduce the possibility of model overfitting.

Finally, we compare the performances of our prediction models with a list of state-of-the-art studies as illustrated in Table 3. However, a direct comparison between the performances of our proposed models and these studies may not be relevant because of the different datasets.

**Table 3:** Comparison between our proposed molecular mutations models and state-of-the art glioma grading models.

Our Proposed Prediction Models 108 LGG (75% training, 25 testing)					Other Models							
Models	Test Performance (using testing sets)			*n*	Study (dataset)	Performance						
	AUC	Sens.	Spec.									
MGMT	0.83 ± 0.04	0.93 ± 0.05	0.73 ± 0.13	11	Kanas *et al*.^50^ (86 GBM patients)	Accuracy			Sens.		Spec.	
						0.736			0.85		0.66	
					Han *et al*.^51^ (159 LGG; 70% training, 15% validation, and 15% testing)	Dataset	AUC	Accuracy	Precision		Recall	
						Test	0.61	0.62	0.67		0.67	
						Validation	0.66	0.67	0.67		0.73	
*IDH*	0.84 ± 0.03	0.90 ± 0.06	0.79 ± 0.09	13	Yu *et al*.^55^ (110 training, 30 independent validation)	Dataset	AUC	Accuracy	Sens.		Spec.	
						Training	0.86	0.80	0.83		0.74	
						Validation	0.79	0.83	0.88		0.67	
					Ding *et al*.^56^ (67 LGG: 48 *IDH* Mutant, 28 *IDH* WT)	AUC	Accuracy	Sens.	Spec.	PPV	NPV	
						0.758	0.793	0.8947	0.60	0.8095	0.75	
1p/19q co-deletion	0.80 ± 0.04	0.75 ± 0.08	0.85 ± 0.06	15	Akkus *et al*.^60^ (159 LGG (252 slices), validation (68 slices), and testing (90 slices)).	Dataset	Accuracy		Sens.		Spec.	
						Testing	0.877		0.933		0.822	
					Van der Voort *et al*.^61^ Training: 284 LGG Testing: 129 LGG	Dataset:	Accuracy	AUC	Sens.	Spec.	F1 score	Precision
						Training	0.698	0.755	0.657	0.721	0.701	0.570
						Testing	0.693	0.723	0.732	0.617	0.697	0.787
*ATRX*	0.70 ± 0.09	0.69 ± 0.06	0.83 ± 0.10	10	Li *et al*.^62^ (95 LGG, 63 training, 32 validation, and 91 external validation	Dataset	Accuracy		AUC	Sens.		Spec.
						Validation	0.938		0.925	0.833		1.00
						External	0.769		0.725	0.571		0.857
*TERT*	0.82 ± 0.04	0.77 ± 0.12	0.86 ± 0.09	14	Wang *et al*.^63^ (39 LGG: 30 *TERT* WT, and 8 *TERT* Mutant)	LOOCV AUC (95% CI) of 0.874 (0.756–0.992)						

Our study on MGMT methylation prediction shows that MGMT methylation correlates with high values of fractal texture features such as histogram entropy of mBm for tumor volume. Entropy measures randomness or uncertainty in the tumor. The analysis reveals that high histogram entropy of mBm associates with the less aggressive methylated MGMT status and carries HR of 0.579 per standard deviation (95%CI, 0.345, 0.969) with a likelihood ratio test *p*-value = 0.035. The study further shows that the size ratio between enhancing tumor and necrosis correlates significantly with un-methylated MGMT, which indicates that the high aggressive MGMT un-methylated LGG, the higher the values of the size ratio. The MGMT methylation prediction study by Kanas *et al*.^50^ for patients with GBM reports the size of the tumor with respect to necrosis as one of the significant features. Our analysis shows that removing the texture features such as fractal and the multi-resolution fractal (of PTPSA, mBm, and Holder Exponent) characterization is significant on the AUC and specificity performance of the MGMT methylation model. The GBM study conducted by Kanas *et al*.^50^ (Table 3) proposes an MGMT prediction model using volumetric, morphological, and locational MR imaging features, respectively. In our study, we use texture features and volumetric features. Moreover, the whole process of the prediction model in our current study including feature extraction is automated, unlike the proposed work by Kanas *et al*.^50^. Another study by Han *et al*.^51^ (Table 3), the authors use a bi-directional convolutional recurrent neural network to predict MGMT methylation status. A major difference between our MGMT prediction model and the method proposed by Han *et al*.^51^ is that our model mainly utilizes quantitative imaging features that can be correlated with tumor biology.

In addition, our *IDH* mutation prediction model indicates that the tumor correlation associates significantly with mutated *IDH* and offers HR of 0.562 per standard deviation with a likelihood ratio test *p*-value = 0.005. In addition, our analysis shows that the complexity of HE of enhancing tumor associates significantly with WT IDH status with HR of 1.553 per standard deviation with a likelihood ratio test *p*-value = 0.008. Complexity is related to the visual information content and the shape of the object. Texture with higher information content and with a large number of edges are considered complex^52^. This outcome is in agreement with another gliomas study by Wang *et al*.^53^ which reports that the enhancement patterns predict the prognosis in *IDH1* mutations in Anaplastic gliomas. Our analysis also shows that the size ratio between enhancing tumor and necrosis is a significant predictor feature of the *IDH* status. This feature is also a significant predictor of MGMT status, which can be explained by the fact that MGMT methylation is associated with *IDH* status as reported by Mukasa *et al*.^54^. In the *IDH* prediction model by Yu *et al*.^55^ (Table 3), the authors use 110 imaging features and SVM to classify *IDH* status with Grade II glioma patients. Even though the dataset we use in the *IDH* mutation prediction is not the same as the dataset is used by Yu *et al*.^55^, the dataset used in our study is more diverse with data from both Grade II and III, and this reflects higher reliability of the performance of the *IDH* status prediction model. A different study by Ding *et al*.^56^ (Table 3) on 76 LGG patients utilizes MR imaging features along with MR spectroscopic data to predict *IDH* mutations using a binary logistic regression model. The authors achieved the best performance when utilizing MR spectroscopic data. When comparing the performance of our *IDH* prediction and the best performance of Ding *et al*.^56^, our model achieves better AUC, sensitivity, and specificity as illustrated in Table 3.

Furthermore, our study on the 1p/19q co-deletion prediction model indicates that the location of the upper-left necrosis bounding box and horizontal coordinate of the necrosis centroid (illustrated in Fig. 4C) are among the most predictive features. This outcome is in agreement with different studies^57–59^ which report that gliomas with 1p/19q co-deletion are associated with the tumor location. In addition, our analysis shows that higher values of histogram entropy of mBm texture of tumor volume are associated significantly with the existence of 1p/19q co-deletion. Moreover, our analysis reveals that removing the texture representation of fractal and multi-resolution fractal from the 1p/19q co-deletion prediction model decreases the AUC and specificity significantly. The test prediction performance of the 1p/19q co-deletion prediction model drops (after removing the fractal features). A study by Akkus *et al*.^60^ (Table 3) with LGG patients (where each patient has 3 MRI slices) proposes 1p/19q co-deletion prediction using a convolutional neural network (CNN). Their method achieves better sensitivity; however, our method achieves slightly better specificity. In addition, Akkus *et al*.^60^ do not consider the global information of the tumor, since their dataset uses only 3 slices of the MRI sequence of each patient as input, not the whole volume of the tumor. Another recent study by van der Voort *et al*.^61^ (Table 3) utilizes MR imaging features along with patients’ age and sex using an SVM classifier to predict 1p/19q co-deletion in LGG patients. The authors use 284 LGG patients for training and another 129 LGG patients for testing. Their analysis reveals that the cranial/caudal location of the tumor is one of the most important features in predicting 1p/19q co-deletion. Comparing the performance of our 1p/19q co-deletion prediction and the performance of van der Voort *et al*.^61^, our 1p/19q co-deletion prediction model outperforms their model as illustrated in Table 3.

Our analysis of *ATRX*-status prediction shows that tumor information measure of correlation imaging feature and histogram mean tumor volume are the most frequently selected features. Higher values of information measure of correlation are associated significantly with WT *ATRX* status. This is in agreement with an *ATRX* mutation prediction study by Li *et al*.^62^ (Table 3) in patients with low-grade glioma, where the authors use MRI texture feature and LASSO regression model. In their model, the information measure of correlation is one of the features that is used to predict *ATRX* mutation. In addition, our analysis shows that the tumor information measure of correlation is one of the most frequent features in the *TERT* model as well. This can be explained by the fact that *ATRX* and *TERT* mutations are mutually exclusive.

The *TERT* prediction analysis shows that tumor information-measure of correlation and upper-left edema bounding box are the most frequently selected features. Higher values of these two features are significantly associated with mutated *TERT* status. The information-measure of correlation assesses the correlation/dependency between two gray-levels using mutual information content. High values of Information measure of correlation are associated with mutated *TERT*. In addition, our analysis suggests that the higher values of Inverse difference moment of HE associates significantly with WT *TERT* and offers HR = 0.612 per standard deviation with a likelihood ratio test, *p*-value = 0.03. Inverse difference moment measures local homogeneity. High values of inverse difference moment of HE tumor predict the less aggressive WT *TERT*. Recently, Wang *et al*.^63^ (Table 3) explore survival prediction and *TERT* mutations in 39 LGG (30 WT, and 9 Mutant) patients and achieve a LOOCV AUC of 0.874 (95% CI: 0.756–0.992). The authors propose a *TERT* prediction model using 24 imaging features selected using Principle Component Analysis (PCA) and classified using the Partial Least Squares (PLS). While their method achieves a slightly higher AUC, the sensitivity, specificity, or the confusion matrix are not provided for such an imbalanced dataset.

**Figure 7:**
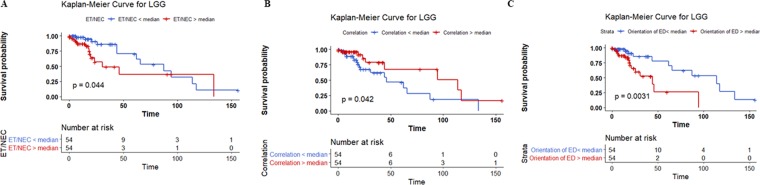
The effect of thresholding (**A**) the size ratio between the enhancing tumor and necrosis, (**B**) tumor correlation, and (**C**) vertical orientation of edema major axis around the median. P-values are computed using the likelihood ratio test.

Overall, our analysis shows that the necrosis location and the necrosis volume-related features are very important (most frequently selected features) in MGMT, *IDH*, and 1p/19q co-deletion prediction. Edema volume-related features are very important in *IDH* and *TERT* prediction models. Fractal features have a significant effect on MGMT, *IDH*, 1p/19q co-deletion, and *ATRX* prediction models. Further analysis on the most frequent features in each prediction model, we notice that the effect of thresholding the value of standardized feature around the median can stratify the 108 cases significantly (log-rank test, *p-*value < 0.05) into two survival groups (Fig. 7A–C). The features and the median survival of each group are:

- the size ratio between the enhancing tumor and necrosis stratifies the 108 cases into two groups with a median survival of 87.4 months vs 30.7 months,
- the correlation of the tumor volume stratifies the 108 cases into two groups with a median survival of 114 months vs 46 months,
- and the vertical orientation of edema major axis stratifies the 108 cases into two groups with a median survival of 114 vs 44 months.

In summary, this study presents molecular prediction model designs from traditional MRI data based on the 2016 update of the WHO classification of LGG of the CNS. Our prediction model performance shows promise when compared to different methods and models in the literature. An association among computed MR imaging features and the molecular mutations LGG was established. The methods discussed in our study are important steps towards non-invasive imaging classification of diffuse LGG based on molecular mutations prior to invasive tissue sampling. In this work for the first time in literature, we hypothesize that fractal and multi-resolution fractal features have an association with molecular prediction. The feature selection using RFS and the subsequent prediction results in Table 1 confirm our hypothesis by showing the efficacy of these fractal features in glioma prediction. Therefore, this work may be considered as a validation of previously hypothesized fractal biomarkers, and, hence, may have potential for generalizability for other types of tumors.
